# Generative adversarial network-based attenuation correction for ^99m^Tc-TRODAT-1 brain SPECT

**DOI:** 10.3389/fmed.2023.1171118

**Published:** 2023-08-15

**Authors:** Yu Du, Han Jiang, Ching-Ni Lin, Zhengyu Peng, Jingzhang Sun, Pai-Yi Chiu, Guang-Uei Hung, Greta S. P. Mok

**Affiliations:** ^1^Biomedical Imaging Laboratory (BIG), Department of Electrical and Computer Engineering, Faculty of Science and Technology, University of Macau, Taipa, Macau SAR, China; ^2^Center for Cognitive and Brain Sciences, Institute of Collaborative Innovation, University of Macau, Taipa, Macau SAR, China; ^3^Department of Nuclear Medicine, Show Chwan Memorial Hospital, Lukong Town, Changhua County, Taiwan; ^4^Department of Neurology, Show Chwan Memorial Hospital, Lukong Town, Changhua County, Taiwan; ^5^Department of Nuclear Medicine, Chang Bing Show Chwan Memorial Hospital, Lukong Town, Changhua County, Taiwan

**Keywords:** deep learning, generative adversarial network, attenuation correction, dopamine transporter SPECT, ^99m^Tc-TRODAT-1

## Abstract

**Background:**

Attenuation correction (AC) is an important correction method to improve the quantification accuracy of dopamine transporter (DAT) single photon emission computed tomography (SPECT). Chang's method was developed for AC (Chang-AC) when CT-based AC was not available, assuming uniform attenuation coefficients inside the body contour. This study aims to evaluate Chang-AC and different deep learning (DL)-based AC approaches on ^99m^Tc-TRODAT-1 brain SPECT using clinical patient data on two different scanners.

**Methods:**

Two hundred and sixty patients who underwent ^99m^Tc-TRODAT-1 SPECT/CT scans from two different scanners (scanner A and scanner B) were retrospectively recruited. The ordered-subset expectation-maximization (OS-EM) method reconstructed 120 projections with dual-energy scatter correction, with or without CT-AC. We implemented a 3D conditional generative adversarial network (cGAN) for the indirect deep learning-based attenuation correction (DL-AC_μ_) and direct deep learning-based attenuation correction (DL-AC) methods, estimating attenuation maps (μ-maps) and attenuation-corrected SPECT images from non-attenuation-corrected (NAC) SPECT, respectively. We further applied cross-scanner training (cross-scanner indirect deep learning-based attenuation correction [cull-AC_μ_] and cross-scanner direct deep learning-based attenuation correction [call-AC]) and merged the datasets from two scanners for ensemble training (ensemble indirect deep learning-based attenuation correction [eDL-AC_μ_] and ensemble direct deep learning-based attenuation correction [eDL-AC]). The estimated μ-maps from (c/e)DL-AC_μ_ were then used in reconstruction for AC purposes. Chang's method was also implemented for comparison. Normalized mean square error (NMSE), structural similarity index (SSIM), specific uptake ratio (SUR), and asymmetry index (%ASI) of the striatum were calculated for different AC methods.

**Results:**

The NMSE for Chang's method, DL-AC_μ_, DL-AC, cDL-AC_μ_, cDL-AC, eDL-AC_μ_, and eDL-AC is 0.0406 ± 0.0445, 0.0059 ± 0.0035, 0.0099 ± 0.0066, 0.0253 ± 0.0102, 0.0369 ± 0.0124, 0.0098 ± 0.0035, and 0.0162 ± 0.0118 for scanner A and 0.0579 ± 0.0146, 0.0055 ± 0.0034, 0.0063 ± 0.0028, 0.0235 ± 0.0085, 0.0349 ± 0.0086, 0.0115 ± 0.0062, and 0.0117 ± 0.0038 for scanner B, respectively. The SUR and %ASI results for DL-AC_μ_ are closer to CT-AC, Followed by DL-AC, eDL-AC_μ_, cDL-AC_μ_, cDL-AC, eDL-AC, Chang's method, and NAC.

**Conclusion:**

All DL-based AC methods are superior to Chang-AC. DL-AC_μ_ is superior to DL-AC. Scanner-specific training is superior to cross-scanner and ensemble training. DL-based AC methods are feasible and robust for ^99m^Tc-TRODAT-1 brain SPECT.

## Introduction

Dopamine transporter (DAT) single photon emission computed tomography (SPECT) is well established and widely used for Parkinson's disease (PD) diagnosis. The current PD diagnosis from DAT SPECT is mainly based on the visual assessment of the decreased striatal uptakes and the asymmetry of left and right striatum uptake for indirect measurement of DAT decrement (1). Iodine ^123^-radiolabeled 2β-carbomethoxy-3β-(4-iodophenyl)-N-(3-fluoropropyl) nortropane (^123^I-FP-CIT) (2) and ^99m^Tc-[2[[2-[[[3-(4-chlorophenyl)-8-methyl-8-azabicyclo[3,2,1]-oct-2-yl]-methyl](2-mercaptoethyl) amino]ethyl]amino]ethane-thiolato(3-)-N2,N2′,S2,S2]oxo-[1R-(exo-exo)])) (99mTc-TRODAT-1) (3) are two common tracers for DAT SPECT, with the former more common in Western countries and the latter more common in Asia. ^123^I-FP-CIT is a European Medicines Agency (EMA) and U.S. Food and Drug Administration (FDA)-approved tracer to differentiate PD from essential tremor. Compared to ^123^I-FP-CIT, ^99m^Tc-TRODAT-1 has a lower binding ratio of the striatum, lower thyroid uptake, and can be produced at a lower cost without a cyclotron. Though the clinical utility of CT-based attenuation correction (AC) (4) is controversial in ^123^I-FP-CIT SPECT (5), CT-based AC has been proven to improve SPECT image quality and quantification accuracy in DAT SPECT (6, 7). In hybrid SPECT/CT systems, CT scans can be used as attenuation maps (μ-maps) for AC in brain SPECT reconstructions. However, many existing SPECT-only systems or recently proposed dedicated brain SPECT images (8) are not integrated with CT scanners. In addition, the extra CT radiation dose in SPECT/CT poses substantial health concerns (9) and is not routinely performed in some centers. Potential mismatches between SPECT and CT images due to the involuntary and voluntary movements of PD patients can also degrade AC performance (10, 11). CT-less AC is thus of significant research and clinical impact for DAT SPECT.

**Figure 1:**
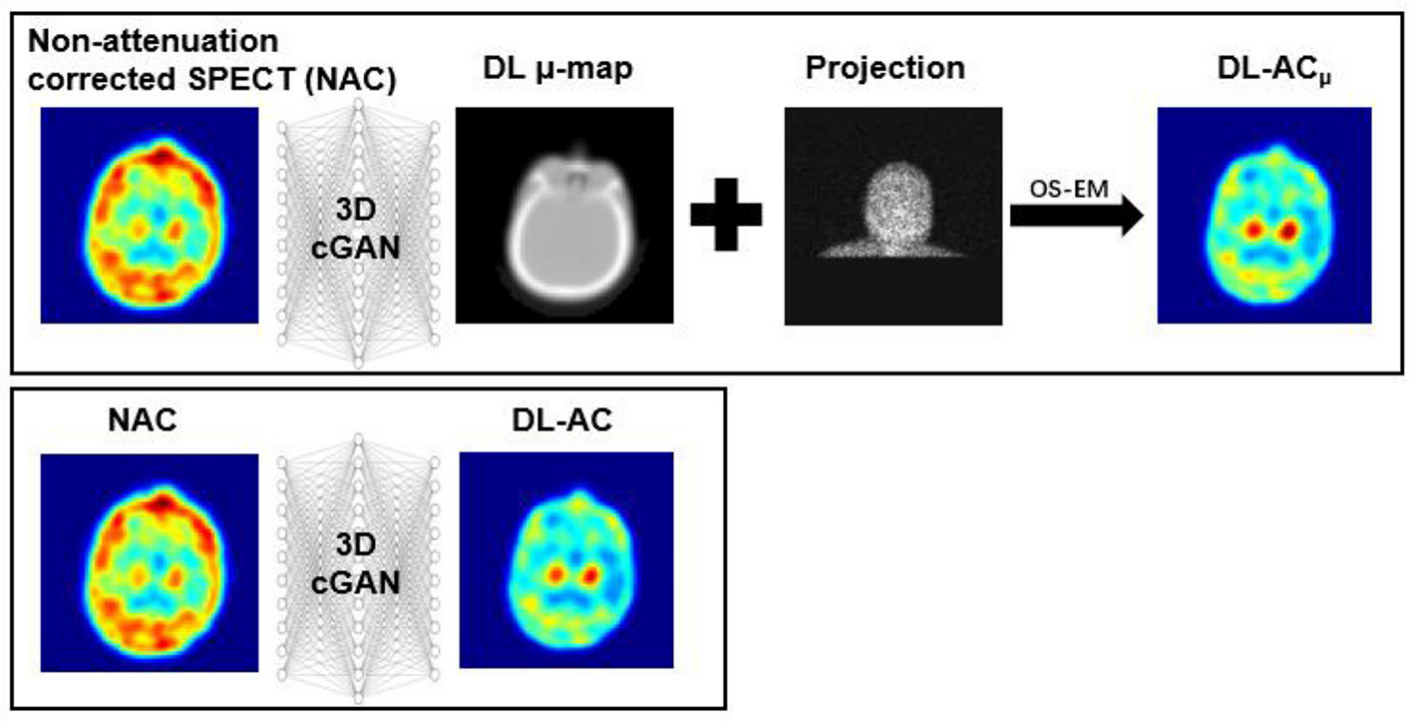
Diagram of DL-AC_μ_ (indirect) and DL-AC (direct) methods.

Chang's AC method is a conventional CT-less AC method for brain SPECT that assumes a uniform attenuation coefficient for the volume of interest (VOI) (12). However, the assumption of a uniform μ-map would introduce estimation errors to AC, especially for bones. Deep learning (DL) methods recently emerged as a promising alternative for SPECT AC (13). Shi et al. (14) and Yang et al. (15) first performed DL-based AC on SPECT. Shi et al. (14) generated μ-maps from non-attenuation-corrected (NAC) SPECT images (indirect deep learning-based attenuation correction [DL-AC_μ_]) using a 3D conditional generative adversarial network (cGAN) for myocardial perfusion (MP) SPECT. Yang et al. (15) and Chen et al. (16) estimated AC MP SPECT images directly from NAC MP SPECT images (direct deep learning-based attenuation correction [DL-AC]) using different deep convolutional neural networks. Chen et al. (17) and Du et al. (18) compared the AC performance of DL-AC and DL-AC_μ_ and demonstrated that indirect estimation of μ-maps is superior to direct estimation of AC SPECT on MP SPECT. Chen et al. (19) further investigated the feasibility of transfer learning-based AC for MP SPECT images from different scanners, tracers, and acquisition protocols. For brain SPECT, Sakaguchi et al. (20) developed a 2D convolutional neural networks (CNN)-based autoencoder for the direct generation of AC from NAC images for brain perfusion SPECT. Murata et al. (21) compared Chang's AC with a 2D autoencoder and U-Net for DL-AC for brain perfusion SPECT. Chen et al. have proposed CNN-based μ-map generation for brain perfusion SPECT (22) and ^123^I-FP-CIT SPECT (23) using NAC SPECT input in simulations, demonstrating improved absolute quantification accuracy. A diagram explaining DL-AC_μ_ and DL-AC is shown in Figure 1. In this study, we implemented a conventional first-order Chang's method and a 3D cGAN for DL-AC and DL-AC_μ_, respectively. We then compared their performance for ^99m^Tc-TRODAT-1 brain SPECT based on clinical data from two scanners in a single center with different acquisition protocols, field-of-view, and voxel sizes.

## Materials and methods

### Clinical dataset

Two hundred and sixty anonymized patients (Table 1) who underwent ^99m^Tc-TRODAT-1 scan in two widely used clinical SPECT/CT systems (Infinia Hawkeye, GE Healthcare, Wauwatosa, WI, USA; Symbia, Siemens Healthineers, Erlangen, Germany) from two affiliated centers were retrospectively recruited under the local ethics approval (SCMH_IRB No: 1110704). Unpaired *t*-test on age and χ^2^ test on gender showed no significant difference between the training-validation dataset and the testing dataset (all ps>0.05). The acquisition protocols of the two scanners are shown in Table 2. CT was acquired for SPECT AC after the SPECT scan. The CT was manually registered to SPECT by the scanner software and converted to the μ-map using a bilinear model (4). No mismatches were observed between the CT and SPECT images in this study.

**Table 1:** Demographic information for the patient study.

	**Scanner A**		**Scanner B**	
Dataset	Training + validation	Testing	Training + validation	Testing
Patient number	100	30	100	30
Age (years)	72.74 ± 13.14 (range: 29 to 98)	71.17 ± 10.28 (range: 49 to 88)	70.88 ± 9.84 (range: 35 to 87)	72.20 ± 7.85 (range: 55 to 85)
*p*-value	0.56		0.50	
Sex	50 male /50 female	18 male/12 female	55 male/45 female	14 male/16 female
*p*-value	0.067		0.096	

**Table 2:** Acquisition protocols for the patient study.

	**Scanner A**	**Scanner B**
Hospital	Chang Bing Show Chwan Memorial Hospital	Show Chwan Memorial Hospital
Model	GE Infinia Hawkeye	SIEMENS Symbia
Injection activity	1,110 MBq	925 MBq
Acquisition time (s/view)	60	45
Collimator type	LEHR	
Primary/scatter window (keV)	126 to 154/114 to 126	126 to 154/109 to 126
Projection number	120 over 360°	90 over 360° 31, 120 over 360° (99)
Reconstruction	OS-EM; 8 iterations × 4 subsets	
	Dual-energy window scatter correction, with or without attenuation correction	
Post-reconstruction filter	3D Gaussian filter	3D Gaussian filter
	σ = 0.8 voxels	σ = 1.2 voxels
Matrix/voxel size (mm)	64 × 64 × 64/4.4181 (30); 128 × 128 × 128/2.761 (100)	128 × 128 × 128/2.6970
CT scan	4-slice, 2.5 mAs,	2-slice, 10 mAs,
	140 kVp, 1.9 pitch,	130 kVp, 1.5 pitch,
	5 mm thickness	3 mm thickness

SPECT projections were reconstructed with dual-energy window scatter correction (24), with or without CT-based AC. The SPECT images with a matrix size of 64 × 64 × 64 and a voxel size of 0.4418 cm/voxel from scanner A were resampled to a matrix size of 128 × 128 × 128 and a voxel size of 0.2761 cm/voxel. We implemented the first-order Chang's method for conventional CT-less AC in brain SPECT. The uniform μ-maps were based on the NAC SPECT brain mask. An intensity threshold of 2 was used to separate the brain from the background. The masks were applied with uniform attenuation coefficients of 0.148 cm^−1^ (25). The 0.148 cm^−1^ attenuation coefficients were then converted to 0.0408 voxel^−1^ for scanner A and 0.0399 voxel^−1^ for scanner B by multiplying with a voxel size of 0.2761 cm/voxel and 0.2697 cm/voxel to get the Chang's method μ-maps, which were used in reconstruction for Chang-AC.

### Conditional generative adversarial network

**Figure 2:**
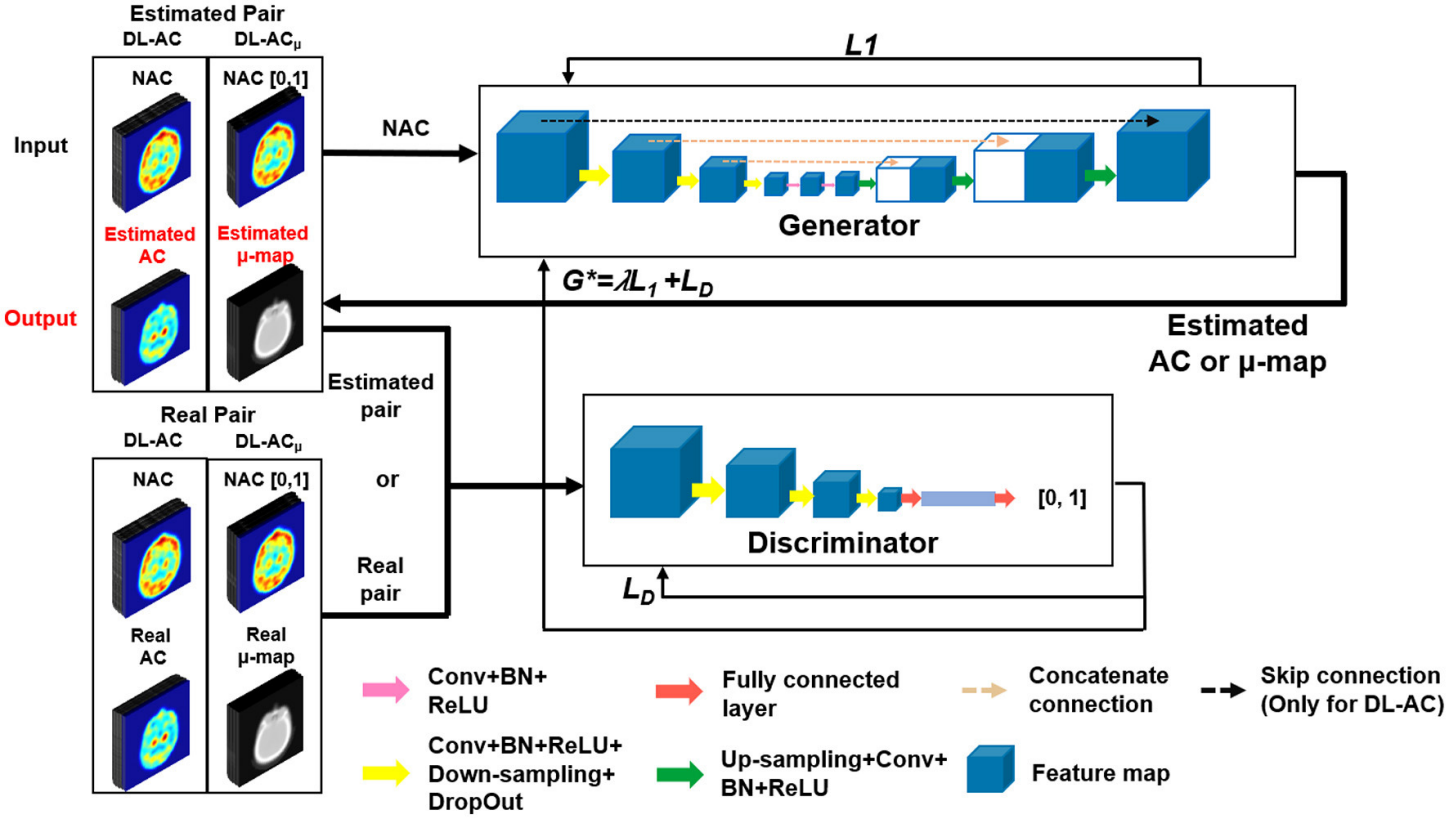
Diagram of 3D cGAN used in this study.

We implemented a 3D cGAN (18, 26) using Tensorflow on an NVIDIA GeForce RTX 3090 GPU with 24 GB RAM. An Adam optimizer was applied using an adaptive learning rate with an initial value of 0.001 and trained for up to 400 epochs. For our cGAN (Figure 2), a U-Net-based generator is trained to generate a realistic μ-map or AC SPECT, while a CNN-based discriminator is trained to differentiate between the true μ-map and the generated μ-map or between the true AC SPECT and the generated AC SPECT. The U-Net-based generator consists of encoder, bottleneck, and decoder layers. Each layer contains a 3 × 3 × 3 convolution, a batch normalization, and a leaky rectified linear unit (LeakyReLU). For encoder layers, a down-sampling layer of 2 × 2 × 2 max pooling was connected to down-sample feature maps. For bottleneck layers, a dropout layer with a 50% dropout rate was used to avoid over-fitting. For decoder layers, the up-sampling layer was used to recover the input image size. Skip connection was applied for DL-AC but not for DL-AC_μ_ due to the large structural difference between SPECT and CT images (18, 27).

*L*_1_ loss was used to train the generator as it can enforce low-frequency correctness and encourage less blurring as compared to the commonly used *L*_2_ (28). The cross-entropy-based discriminator loss (*L*_*D*_) was used to train the discriminator. The discriminator loss combined with generator loss (*L*_1_) was used to train the generator.

The objective function of cGAN can be expressed as follows:


(1) $$\begin{array}{l} G^{{\;}^{*}}=\;\arg\underset{G}{\min}\underset{D}{\max}V_{L_{D}}\left(G,D\right)+\;\lambda V_{L1}\left(G\right) \end{array}$$

where λ is an adjustable parameter (28) [λ = 20 in this study (14)] used to control the balance between objective function *V*_*L*_*D*__(*G, D*) and *V*_*L*1_(*G*).

Whole images instead of patches were used as training input to provide more information on the 3D patient contour (18). The patient dataset of each scanner was divided into 90, 10, and 30 for training, validation, and testing, respectively. Data augmentation by horizontal and vertical flips was used to enhance the training dataset, i.e., a total of 270 datasets were used for training. As supervised learning methods, the AC SPECT and NAC SPECT images were paired to train DL-AC, while the μ-maps and NAC SPECT images were paired to train DL-AC_μ_. The input NAC SPECT images for DL-AC_μ_ were normalized to a range of [0, 1], better matching the range of attenuation coefficients from the μ-maps. Network hyperparameters were chosen based on our previous DL-based AC for MP SPECT, i.e., 3 layers and 48 feature maps for DL-AC_μ_ and 2 layers and 48 feature maps for DL-AC (18).

To confirm the robustness of the DL-based AC methods, we applied cross-scanner training (cross-scanner indirect deep learning-based attenuation correction [cDL-AC_μ_] and cross-scanner direct deep learning-based attenuation correction [cDL-AC]), i.e., testing data from scanner A based on a model trained from scanner B and vice versa. We also merged the datasets from two scanners for ensemble training (ensemble indirect deep learning-based attenuation correction [eDL-AC_μ_] and ensemble direct deep learning-based attenuation correction [eDL-AC]). The same data augmentation technique used in scanner-specific training was applied in cross-scanner and ensemble training.

### Data analysis

Normalized mean square error (NMSE) and structural similarity index (SSIM) for the whole brain were computed on the NAC, Chang's AC, and DL-based AC images as compared to the CT-AC SPECT images. The count profile across the striatum region was also drawn to measure the count distribution of different AC methods.

**Figure 3:**
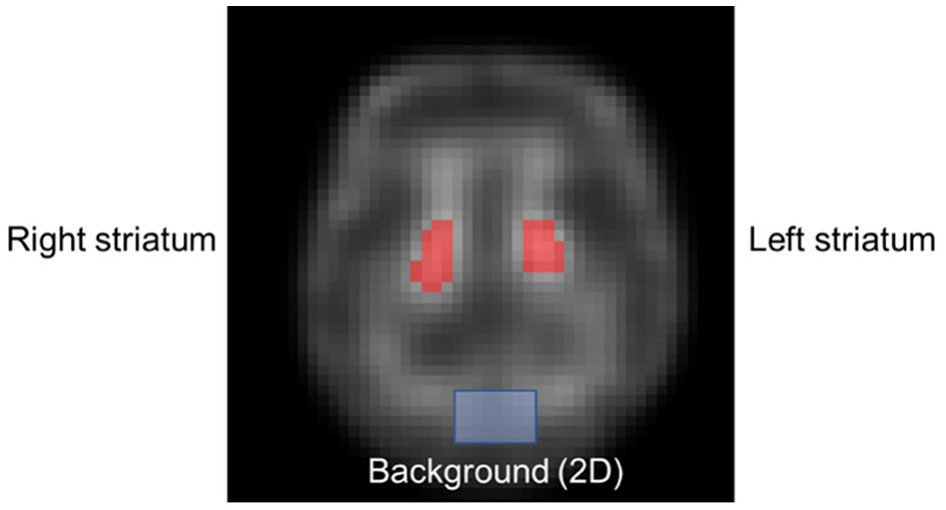
Sample delineated striatum ROIs (red mask) and background (blue mask).

Specific uptake ratio (SUR) (Equation 2) of the whole striatum to the background regions and asymmetry index (%ASI) (Equation 3) of the left and right striatum were calculated based on the striatal VOIs delineated by an experienced nuclear medicine physician (Figure 3). The 2D striatal regions of interest (ROIs) were delineated slice by slice, stacking to form a 3D VOI. For the background, a 2D ROI (10 pixels × 6 pixels) in the cerebellum region was chosen, excluding ventricular regions. Bland–Altman plots were applied to SUR and %ASI results to evaluate the potential difference between different AC methods as compared to CT-AC. A paired *t*-test was performed on NMSE, SSIM between different DL-based methods, and on SUR and %ASI each between AC method and CT-AC. Bonferroni correction was applied for tests with multiple comparisons.


(2) $$\begin{array}{l} SUR=\;\frac{MeanCounts_{striatum}-MeanCounts_{background}}{MeanCounts_{background}} \end{array}$$

(3) $$\begin{array}{l} \%ASI=\;|\frac{SUR_{left\;striatum}-SUR_{right\;striatum}}{SUR_{left\;striatum}+SUR_{right\;striatum}}|\;\times 100\% \end{array}$$

## Results

**Figure 4:**
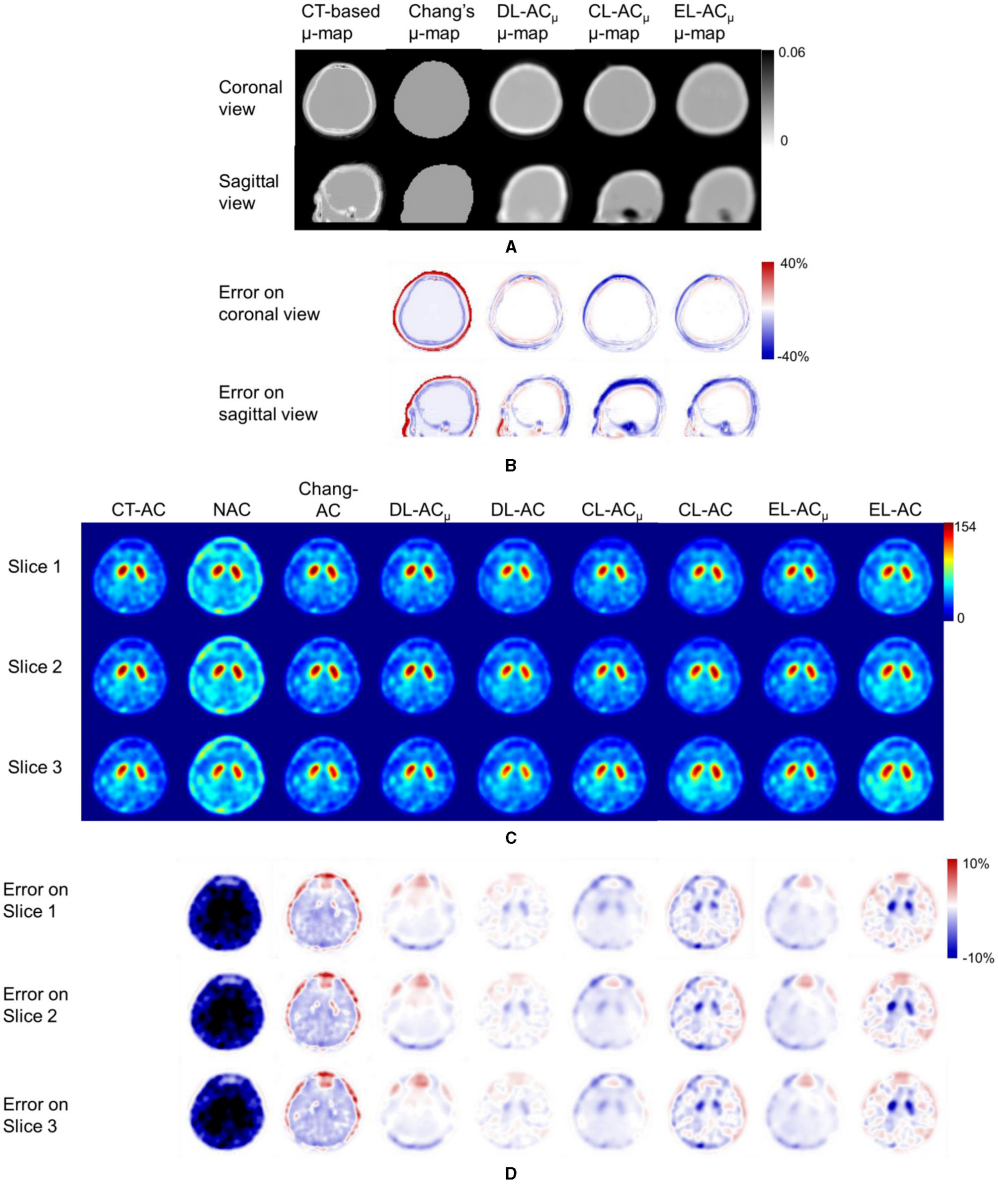
Representative image results of a 38-year-old male patient on scanner A. **(A)** Sample μ-maps generated by different methods. **(B)** Corresponding error maps of different μ-maps as compared to CT-based μ-map. **(C)** Sample axial slices with the highest striatum uptake from different AC SPECT images. **(D)** Corresponding error maps of different AC methods as compared to CT-AC.

**Figure 5:**
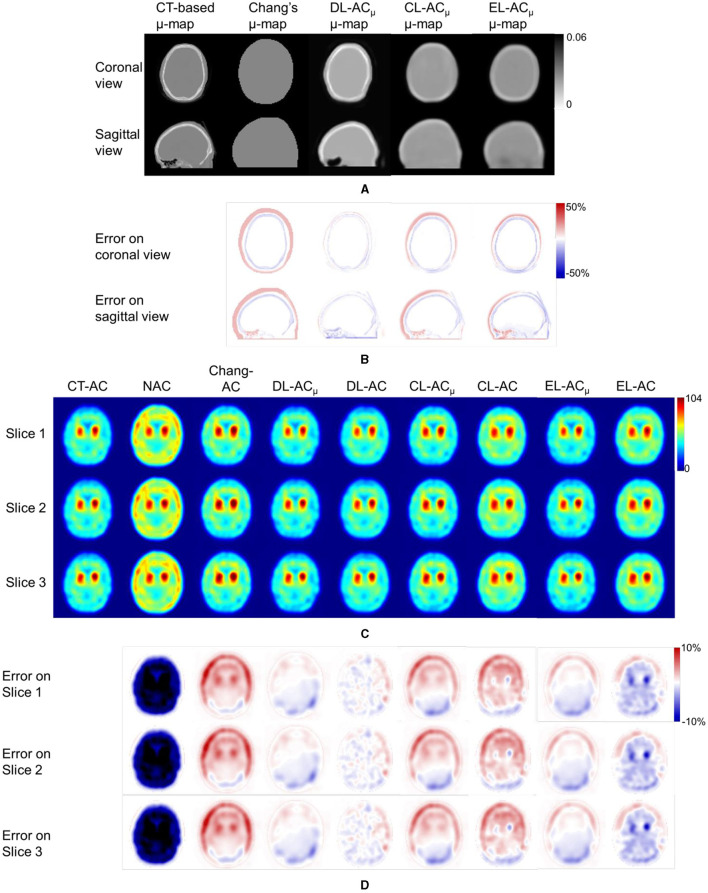
Representative results of a 71-year-old female patient on scanner B. **(A)** Sample μ-maps generated by different methods. **(B)** Corresponding error maps of different μ-maps as compared to CT-based μ-map. **(C)** Sample axial slices with the highest striatum uptake from different AC SPECT images. **(D)** Corresponding error maps of different AC methods as compared to CT-AC.

**Figure 6:**
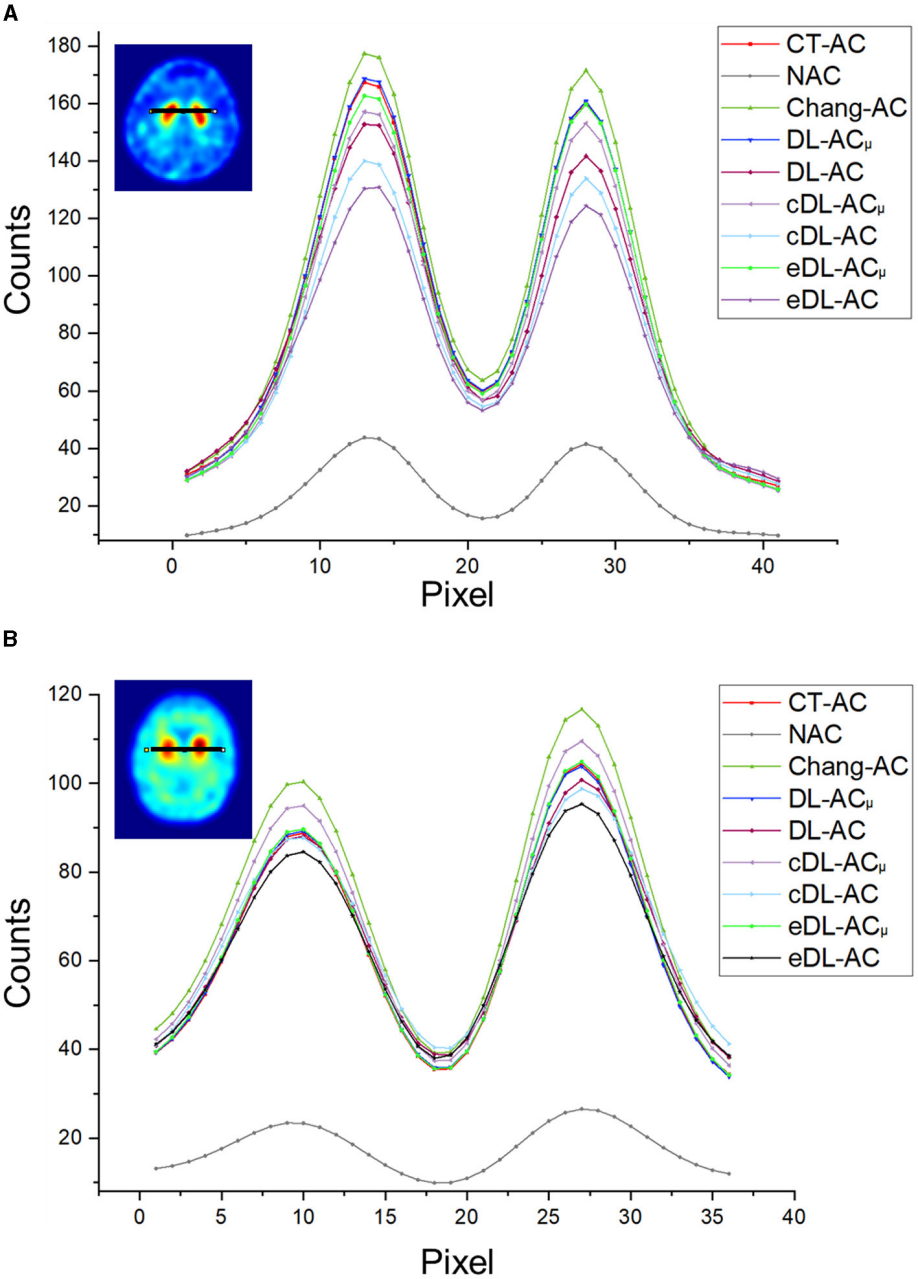
Count profiles of different AC methods of **(A)** a 38-year-old male patient on scanner A from Figure 4 and **(B)** a 71-year-old female patient on scanner B from Figure 5.

Figures 4A, B shows sample Chang's μ-map, DL-AC_μ_, cDL-AC_μ_, and eDL-AC_μ_ generated μ-maps of scanner A and corresponding error maps using CT-based μ-map as reference. The brain contours are well recovered, while the bony structures could be better restored for DL-AC_μ_. Figures 4C, D shows SPECT images of different AC methods and corresponding error maps using CT-AC SPECT as a reference. Three axial slices containing the highest striatum counts are displayed for comparison. All DL-based AC methods show improved image quality as compared to NAC and Chang-AC, while DL-AC_μ_ is better than DL-AC from a visual assessment based on the error maps. The errors are increased by cross-scanner and ensemble training, while c/eDL-AC_μ_ has fewer errors than the corresponding c/eDL-AC. Figures 5A, B shows the sample results of Chang's μ-maps, different DL-AC_μ_ generated μ-maps, and SPECT images of different AC methods on scanner B. The results are similar to scanner A. Both DL-based AC methods are better than Chang-AC and NAC, while DL-AC_μ_ is better than DL-AC. Though the cross-scanner and ensemble training DL-based methods show performance degradation compared to scanner-specific training, they still show fewer errors than Chang-AC. The 2D count profiles of 40 pixels across the striatum region by different AC methods for the same patients are shown in Figure 6. The profiles of all DL-based AC methods are much closer to CT-AC compared to Chang-AC and NAC, while the profile of DL-AC_μ_ is also less deviated from CT-AC as compared to DL-AC. eDL-AC_μ_ has errors between DL-AC_μ_ and DL-AC, followed by cDL-AC_μ_, cDL-AC, and eDL-AC.

The NMSE and SSIM results of the two scanners are shown in Tables 3, 4. For μ-maps, DL-AC_μ_ achieves the lowest NMSE and highest SSIM (all ps < 0.001), followed by eDL-AC_μ_ and cDL-AC_μ_ for both scanners. All DL-based methods have generated better μ-maps than Chang's μ-maps for both scanners. For SPECT images, the NMSE of all AC methods is lower than NAC. All DL-based AC methods are better than Chang-AC. DL-AC_μ_ has a significantly lower NMSE than DL-AC (*p* < 0.05) for both scanners. The NMSE values of eDL-AC_μ_ and cDL-AC_μ_ are lower than eDL-AC and cDL-AC, respectively. The SSIM follows the same trend as the NMSE. The SUR and %ASI results are shown in Table 5. There is no significant difference between DL-AC_μ_/DL-AC and CT-AC (p > 0.05) on SUR for both scanners. There is also no significant difference between DL-AC_μ_/DL-AC/eDL-AC_μ_/cDL-AC_μ_ and CT-AC (*p* > 0.05) on %ASI for both scanners. Cross-scanner and ensemble training increase the errors on SUR and %ASI scanner-specific training.

**Table 3:** NMSE and SSIM measurements on μ-maps generated by different methods on 30 tested patients of scanner A and 30 tested patients of scanner B.

**Scanner**	**A**		**B**	
Metric	NMSE	SSIM	NMSE	SSIM
Chang-AC	0.2933 ± 0.0383	**0.9766** ± 0.0072	**0.2824** ± 0.0306	**0.9718** ± 0.0064
DL-AC_μ_	0.0348 ± 0.0133	0.9935 ± 0.0127	0.0591 ± 0.0185	0.9917 ± 0.0042
cDL-AC_μ_	0.1427 ± 0.0305	0.9856 ± 0.0124	0.1345 ± 0.0345	0.9849 ± 0.0051
*p*-value	2.11 × 10^−15^	4.74 × 10^−17^	6.91 × 10^−24^	4.98 × 10^−15^
eDL-AC_μ_	0.0532 ± 0.0158	0.9917 ± 0.0137	0.0702 ± 0.0215	0.9916 ± 0.0034
*p*-value	1.42 × 10^−8^	2.97 × 10^−23^	8.52 × 10^−7^	2.66 × 10^−8^

**Table 4:** NMSE and SSIM measurements on SPECT images with different AC methods on 30 tested patients of scanner A and 30 tested patients of scanner B.

**Scanner**	**A**		**B**	
Metric	NMSE	SSIM	NMSE	SSIM
NAC	0.4495 ± 0.0193	0.9187 ± 0.0977	0.3900 ± 0.0338	0.9038 ± 0.0170
Chang-AC	0.0406 ± 0.0445	0.9415 ± 0.0144	0.0579 ± 0.0146	0.9562 ± 0.0081
DL-AC_μ_	0.0059 ± 0.0035	0.9982 ± 0.0036	0.0055 ± 0.0034	0.9973 ± 0.0011
DL-AC	0.0099 ± 0.0066	0.9974 ± 0.0016	0.0063 ± 0.0028	0.9938 ± 0.0017
*p*-value	1.90 × 10^−4^	1.84 × 10^−3^	3.68 × 10^−4^	2.34 × 10^−19^
cDL-AC_μ_	0.0253 ± 0.0102	0.9913 ± 0.0136	0.0235 ± 0.0085	0.9932 ± 0.0025
cDL-AC	0.0369 ± 0.0124	0.9900 ± 0.0027	0.0349 ± 0.0086	0.9930 ± 0.0019
*p*-value	3.80 × 10^−4^	1.07 × 10^−3^	4.46 × 10^−7^	0.11
eDL-AC_μ_	0.0098 ± 0.0035	0.9935 ± 0.0027	0.0115 ± 0.0062	0.9963 ± 0.0017
eDL-AC	0.0162 ± 0.0118	0.9965 ± 0.0024	0.0117 ± 0.0038	0.9950 ± 0.0017
*p*-value	6.92 × 10^−27^	2.11 × 10^−2^	0.67	1.24 × 10^−14^

**Table 5:** SUR and %ASI measurements on SPECT images with different AC methods on 30 tested patients of scanner A and 30 tested patients of scanner B.

**Scanner**	**A**		**B**	
Metric	SUR (*p*-value)	%ASI (*p*-value)	SUR (*p*-value)	%ASI (*p*-value)
CT-AC	0.71 ± 0.33	11.75 ± 15.52	0.67 ± 0.21	9.01 ± 7.37
NAC	0.40 ± 0.18 (2.47 × 10^−16^)	19.88 ± 23.59 (8.92 × 10^−4^)	0.49 ± 0.22 (2.65 × 10^−11^)	12.83 ± 12.43 (1.24 × 10^−2^)
Chang-AC	0.80 ± 0.38 (2.75 × 10^−2^)	10.17 ± 15.90 (8.13 × 10^−3^)	0.76 ± 0.26 (5.28 × 10^−16^)	8.13 ± 6.54 (1.85 × 10^−3^)
DL-AC_μ_	0.68 ± 0.37 (0.13)	12.54 ± 14.93 (0.31)	0.68 ± 0.21 (0.12)	9.01 ± 7.17 (0.99)
DL-AC	0.77 ± 0.35 (4.68 × 10^−5^)	13.01 ± 20.46 (0.07)	0.62 ± 0.20 (0.08)	8.83 ± 8.01 (0.87)
cDL-AC_μ_	0.59 ± 0.40 (1.70 × 10^−9^)	12.88 ± 15.63 (0.11)	0.74 ± 0.22 (1.82 × 10^−9^)	8.90 ± 7.43 (0.91)
cDL-AC	0.55 ± 0.38 (3.34 × 10^−10^)	13.44 ± 21.58 (2.65 × 10^−11^)	0.70 ± 0.22 (2.11 × 10^−2^)	8.54 ± 7.64 (5.70 × 10^−2^)
eDL-AC_μ_	0.64 ± 0.35 (0.09)	12.65 ± 15.19 (0.55)	0.68 ± 0.21 (0.13)	9.04 ± 8.02 (0.96)
eDL-AC	0.58 ± 0.41 (9.55 × 10^−9^)	13.25 ± 21.03 (2.50 × 10^−5^)	0.53 ± 0.22 (3.87 × 10^−9^)	10.97 ± 10.31 (3.87 × 10^−2^)

**Figure 7:**
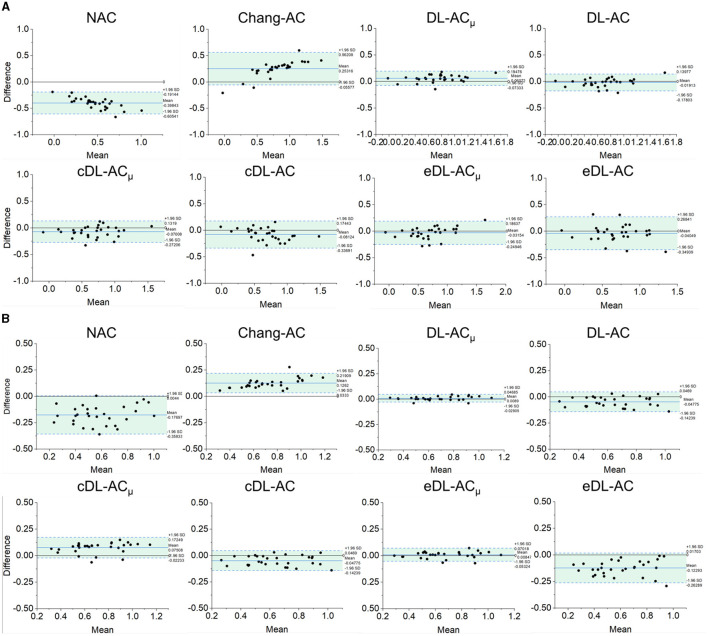
Bland–Altman plots of SUR results of different AC methods, using CT-AC as the reference of **(A**) scanner A and **(B)** scanner B (the dotted lines indicate 95% CI, and the blue lines indicate the mean values).

**Figure 8:**
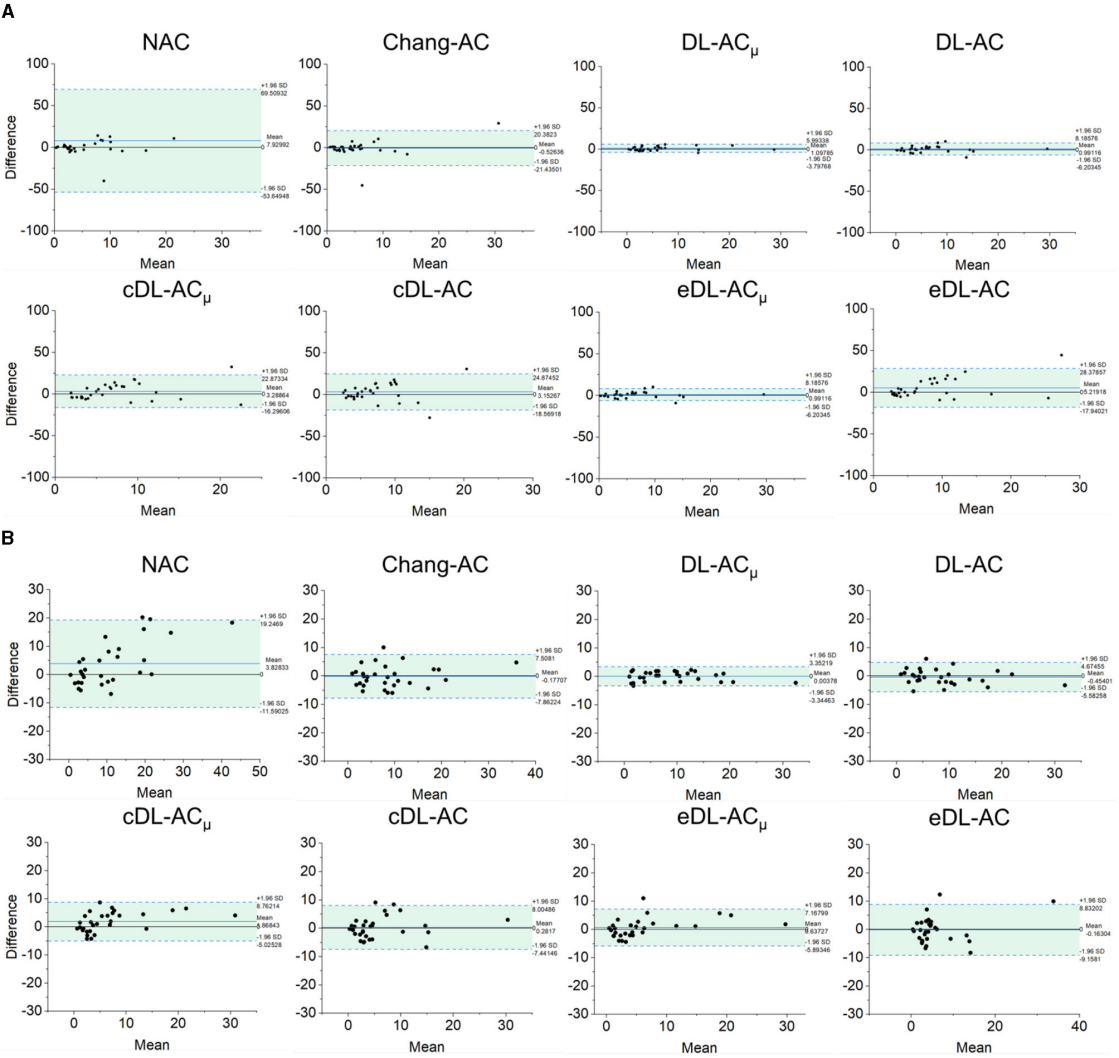
Bland–Altman plots of %ASI results of different AC methods, using CT-AC as the reference of **(A)** scanner A and **(B)** scanner B (the dotted lines indicate 95% CI, and the blue lines indicate the mean values).

The Bland–Altman plots of SUR processed by NAC, Chang-AC, and DL-based methods using CT-AC as a reference are shown in Figure 7. NAC shows lower SUR values (mean difference of −0.3984 for scanner A and −0.1770 for scanner B) than CT-AC. Chang-AC shows an overestimated attenuation (mean difference of +0.2532 for scanner A and +0.1262 for scanner B). All DL-based AC methods have a narrower distribution than NAC and Chang-AC, except cDL-AC and eDL-AC. Both DL-AC_μ_ and DL-AC have similar SUR values to CT-AC. DL-AC_μ_ shows a narrower distribution compared to DL-AC with a smaller standard deviation, i.e., 95% confidence interval (CI) of [−0.0733, 0.1948] vs. [−0.1780, 0.1398] for scanner A and [−0.0291, 0.0469] vs. [−0.1424, 0.0469] for scanner B. Figure 8 shows the Bland–Altman plots of the %ASI results, which are similar to those of SUR. The 95% CI with CT-AC for NAC, Chang-AC, DL-AC_μ_, and DL-AC are [−53.65, 69.51], [−21.44, 20.38], [−3.798, 5.993], [−6.203, 8.186] for scanner A and [−11.59, 19.25], [−8.300, 7.009], [−3.345, 3.352], [−5.583, 4.675] for scanner B, respectively. For cross-scanner and ensemble training, the 95% CI with CT-AC for cDL-AC_μ_, cDL-AC, eDL-AC_μ_, and eDL-AC are [−16.30, 22.87], [−18.57, 24.87], [−6.203, 8.186], [−17.94, 28.38] for scanner A, and [−5.025, 8.762], [−7.441, 8.005], [−5.893, 7.168], [−9.158, 8.832] for scanner B.

## Discussion

Our study is the first DL-based AC study using clinical ^99m^Tc-TRODAT-1 brain SPECT data. In addition, we recruited patient data from two scanners with different acquisition protocols, field-of-view, and voxel sizes to show the robustness of DL-based AC methods on different scanners. Despite slight differences between the two scanners, e.g., brain orientation and field-of-view, spatial resolution, and CT slice thickness, their results showed similar trends for different AC methods. Murata et al. (21) demonstrate that 2D autoencoder and U-Net-based direct DL-AC are better than NAC and Chang's AC for brain perfusion SPECT. Chen et al. (23) suggest that CNN-estimated μ-map could be a promising substitute for CT-based μ-map for ^123^I-FP-CIT scans. Our results are consistent with theirs in that DL-based AC is better than NAC and Chang's method. We further demonstrate that the generation of μ-map is superior to the direct generation of AC SPECT for ^99m^Tc-TRODAT-1 SPECT.

Furthermore, to the best of our knowledge, there is also no comparison of Chang's method with direct and indirect DL-based AC methods for DAT SPECT. As expected, the results show that Chang-AC has generally better image quality and quantitative results than NAC. However, the assumption of uniform attenuation coefficients over the whole brain is obviously problematic for brain SPECT, as attenuation coefficients for skull bone (0.21~0.27 cm^−1^), soft tissues (0.14~0.15 cm^−1^), and nasal cavity or mouth (~0 cm^−1^) are quite different based on the measurements on our data, consistent with previous measurements (4). There are also potential errors in the estimation of the brain contour on SPECT-reconstructed images in Chang's method. In this study, Chang's μ-maps are ~2–4 voxels larger than the original CT-based μ-maps in three dimensions (Figure 4). This is probably attributed to the inherent limitation of the use of thresholding method to generate Chang's μ-maps (25). A larger μ-map would lead to an overestimation of attenuation outside the skull region, i.e., the red rim in Figures 4B, 5B. Thus, DL-based methods could provide a promising alternative.

For DL-AC_μ_, the μ-maps can generally be estimated well for both scanners. That could be attributed to NAC SPECT images providing a rough estimate of the brain contour based on the background uptake. For all quantitative indices, all AC methods significantly improve the image quality as compared to NAC, while both DL-based methods are significantly better than Chang-AC (Figures 4–8). The use of a DL-generated μ-map for the AC purpose outperforms a direct DL-AC approach, even with cross-scanner and ensemble training, showing better robustness of indirect DL-AC_μ_ than direct DL-AC, which is consistent with our previous MP SPECT study (18).

Although the cross-scanner and ensemble training models have better NMSE and SSIM than Chang-AC, their performance is still inferior to scanner-specific training. This could be caused by the differences between the two scanners' data, e.g., voxel size, injection dose, acquisition time, and patient positioning, leading to slightly different SPECT and CT image characteristics. Transfer learning aims to address this problem by fine-tuning the pre-trained model with a small dataset from the target scanner (19). This study is beyond the scope of this study and is ongoing in our group (29).

The SUR and %ASI are highly related to the clinical diagnosis of PD by the detection of decrement in DAT and asymmetry uptake in the left and right striatum. NAC has a much lower SUR than other AC methods, as counts are substantially attenuated toward the center of the brain, where the striatum is located. Chang-AC shows an overestimated SUR compared to CT-AC, which has been reported by previous studies (25, 30), yet it is still better than NAC. Thus, NAC may lead to a false positive, and Chang-AC may lead to a false negative diagnosis. All AC methods have better SUR and %ASI results than NAC. For DL-based methods, the SUR and %ASI values are much closer to CT-AC compared to Chang-AC except for cross-scanner-tested cDL-AC and ensemble-trained eDL-AC, indicating an improved count recovery in both the striatal region and background. DL-AC_μ_ is the best AC method for SUR and %ASI. For (c/e)DL-AC_μ_, we have applied normalization to SPECT image intensity, which is not applied on (c/e)DL-AC to keep the counts invariable. Our proposed DL-based AC can improve quantitative accuracy, image quality, and clinical diagnosis accuracy of DAT SPECT, reducing the radiation dose (31, 32), the additional scan time of CT scans, and potential mismatches concern between SPECT and CT images for AC and providing a CT-less AC option for SPECT without integrated CT.

There are certain limitations to this study. Involuntary head motion is commonly observed in patients with neurodegenerative disease (33). It may lead to a mismatch between SPECT and CT data (34–36), which could be reduced by registration (37) and motion-tracking methods (38). Previously, we showed that this mismatch compromises DL-based AC performance, but it is still better than NAC in MP SPECT (18). We expect a similar result for DL-based AC in DAT SPECT.

## Conclusion

This is the first clinical evaluation of DL-based AC methods for DAT SPECT. Both DL-based methods improve image quality and quantitative accuracy as compared to Chang-AC and NAC. DL-AC_μ_ is consistently better than DL-AC on clinical patient data on two scanners with different acquisition protocols and post-processing parameters.

## New knowledge gained

Deep learning-based attenuation correction (AC) is feasible for DAT SPECT. Indirect generation of attenuation maps is better and more robust than direct generation of attenuation-corrected SPECT images from non-attenuation-corrected SPECT images and Chang's AC for DAT SPECT.

## Data availability statement

Patient data are not allowed to be shared with the public due to patient confidentially. Requests to access the datasets should be directed to P-YC (paiyibox@gmail.com) and G-UH (106143@gmail.com).

## Ethics statement

The studies involving human participants were reviewed and approved by Institutional Review Board of Chang Bing Show Chwan Memorial Hospital (IRB number: 1110704). The written consent was waived due to the retrospective nature of this study.

## Author contributions

YD and GM were the primary authors of the manuscript. YD was mainly responsible for data processing and analysis. HJ, ZP, and JS were mainly responsible for data analysis and manuscript revision. C-NL was mainly responsible for data collection. HJ, C-NL, P-YC, and G-UH were responsible for clinical interpretation. GM and G-UH were responsible for data interpretation and study integration. All authors contributed to the article and approved the submitted version.
